# Evaluation of direct-fed microbials as an alternative to ionophores and non-ionophore additives on *in vitro* ruminal fermentation, gas production kinetics, and digestibility in beef cattle’s forage-based diets

**DOI:** 10.1093/tas/txaf148

**Published:** 2025-11-04

**Authors:** Edjane Pereira da Silva, Bruno Ieda Cappellozza, Joanis Tilemahos Zervoudakis, Filipe Araújo Canêdo Mendonça, Rafael Bonfim Fernandes, Luciano da Silva Cabral, Rosemary Lais Galati, Mozart Alves Fonseca, Nelcino Francisco de Paula

**Affiliations:** Faculty de Agronomy and Animal Science, Federal University of Mato Grosso, Cuiabá, MT 78060-900, Brazil; Novonesis, Lyngby 2800, Denmark; Faculty of Veterinary Medicine, Federal University of Mato Grosso, Cuiabá, MT 78060-900, Brazil; Phibro Animal Health Corporation, Campinas, SP 13025-170, Brazil; Faculty de Agronomy and Animal Science, Federal University of Mato Grosso, Cuiabá, MT 78060-900, Brazil; Faculty de Agronomy and Animal Science, Federal University of Mato Grosso, Cuiabá, MT 78060-900, Brazil; Faculty de Agronomy and Animal Science, Federal University of Mato Grosso, Cuiabá, MT 78060-900, Brazil; Department of Animal and Range Sciences, New Mexico State University, Clayton Livestock Research Center, Clayton, NM 88415, United States; Faculty de Agronomy and Animal Science, Federal University of Mato Grosso, Cuiabá, MT 78060-900, Brazil

**Keywords:** *Bacillus spp.*, beef cattle, digestibility, fiber, pasture

## Abstract

Two studies were conducted to evaluate the effects of a *Bacillus*-based direct-fed microbial (**DFM**) compared to ionophores and non-ionophore additives on *in vitro* ruminal fermentation parameters, gas production kinetics, as well as dry matter and fiber digestibility using two forage-based diets (medium- and low-quality tropical forages). For Exp. 1, *Urochloa brizantha* cv. Marandu (CP = 9.64%) was used as the medium-quality substrate (MF), alone or in combination with an energy-protein supplement (EPS), hereafter referred to as MF and MF-S, respectively. These substrates were incubated in triplicate, and six treatments were evaluated: Control (Con**—**no additive); two DFM levels: DFM1x (1.9 mg), and DFM5x (9.5 mg); Monensin (Mon; 20 ppm); Narasin (Nar; 13 ppm); and Flavomycin (Flavo; 4 ppm). In Exp. 2, *U. brizantha* cv. Marandu (CP = 3.0%) was used as the low-quality substrate (LF), alone or in combination with EPS, and defined as LF and LF-S, respectively. The additive treatments were the same as described in Exp. 1. In both experiments, an *in vitro* gas production (GP) system was used in four consecutive 96-h fermentation runs. The average values obtained from three bottles within each incubation were considered as the experimental unit. The data from each experiment were analyzed using a 2 × 6 factorial design. In Exp. 1, there were no significant interactions between substrates and feed additives (*P* > 0.05) for GP, kinetic parameters, and digestibility. DFM1x and DFM5x and Flavo increased (*P* < 0.05) GP in the initial hours of incubation, while Mon and Nar reduced it compared to Con (*P* < 0.01). The rate of digestion for the first pool (K1) was higher for Nar vs. DFM1x, DFM5x, Mon, and Flavo, but did not differ from Con (*P* < 0.01). Relative to other treatments, Mon reduced K1 (*P* < 0.01), and Mon and Nar reduced the rate of digestion for the second pool (K2) (*P* < 0.01). Overall, compared with other treatments, Mon and Nar reduced nutrient digestibility (*P* < 0.01) and acetate: propionate ratio (*P* < 0.01). In Exp. 2, substrate × additive interactions were observed for GP (*P* < 0.05). DFM1x increased (*P* < 0.01) GP compared with Con when substrate was LF, but did not differ (*P* > 0.05) from Con when LF-S was used. Mon and Nar reduced (*P* < 0.05) GP, rate digestion, and increased (*P* < 0.05) lag time compared to Con, DFM1x, DFM5x, and Flavo. Digestibility was not affected (*P* > 0.05) by DFM, but ionophores decreased it (*P* < 0.01) compared to Con. Total VFA did not differ (*P* = 0.11) among treatments, but Mon and Nar reduced (*P* = 0.02) acetate compared with Con and DFM1x and increased (*P* < 0.01) propionate compared with other treatments. These findings suggest, given increasing scrutiny of use of antibiotics and potential antimicrobial resistance uprise, DFM may be a viable alternative to ionophores without impairing ruminal fermentation.

## Teaser

Naturally occurring *Bacillus* bacteria may boost rumen fermentation in cattle fed forage diets, offering a promising natural alternative to conventional chemical feed additives supplementation.

## Introduction

In tropical regions, such as Brazil, cattle production on pasture is challenging due to the seasonal variations in forage availability and nutritional quality of the forage throughout the year. During both the dry and rainy seasons, the forage available for grazing animals should not be considered a balanced diet, as its nutritional characteristics often fail to meet the adequate requirements for optimal animal performance (Almeida et al. 2022; Detmann et al. 2014). The limited amount and availability of crude protein (CP) and high indigestible neutral detergent fiber (NDF) in tropical forages have been recognized as a critical threshold for adequate microbial growth in forage-based diets (Detmann et al. 2014; Leng 1990; Franco et al. 2017). In fact, Almeida et al. (2022) observed that forage CP and CP intake from a supplement were positively correlated with the performance of grazing beef cattle.

Additionally, feed additives have been used in beef cattle systems as an important nutritional tool to enhance productivity (Duffield et al. 2012; Tedeschi et al. 2003). Numerous studies have demonstrated the effectiveness of feed additives in modulating ruminal fermentation dynamics (Hook et al. 2022; Tedeschi et al. 2003), thereby improving feed efficiency in growing and finishing beef cattle (Duffield et al. 2012), preventing rumen acidosis and liver abscesses (McAllister et al. 2020), optimizing health of the beef cattle herd (Azzaz et al. 2015; Lemos et al. 2016; Marques and Cooke 2021), enhancing performance (Morris et al. 1990), and reducing dietary gross energy losses through methane (Appuhamy et al. 2013). However, most of this research was conducted in feedlot cattle fed high-concentrate diets. In addition, studies with feed additives in forage-based diets were carried out with forage protein content above 7% (Limede et al. 2021; Polizel et al. 2020). Bretschneider et al. (2008) suggested that the forage quality may influence the pattern of response to ionophore additives.

Recently, concerns have emerged regarding the use of ionophores, which have been banned from animal production in Europe (Edwards et al. 2005), due to their potential role in promoting antimicrobial resistance (Terry and Beauchemin 2025) and residual and long-term effects (Pasqualino et al. 2020; Soares et al. 2021). As a result, there has been growing interest in feed additives considered more natural and safer (Jouany and Morgavi 2007; Terry and Beauchemin 2025). Therefore, attention has turned to the use of direct-fed microbials (DFM) in beef and dairy cattle diets, as potential alternatives to antibiotic feed additives (Krehbiel et al. 2003; Luise et al. 2022). Direct-fed microbials, such as *Bacillus subtilis* and *B. licheniformis* can directly produce enzymes and/or stimulate the growth of symbiotic bacteria (Luise et al. 2022, Schallmey et al. 2004). Consequently, greater *in vitro* and *in situ* dry matter and neutral detergent fiber digestibility have been reported in previous studies (Cappellozza et al. 2023; Limede et al. 2025; Pan et al. 2022). However, additional data is required in production settings using tropical forages.

It was hypothesized that a *Bacillus*-based DFM (*B. licheniformis* 809 and *B. subtilis* 810) would modulate ruminal fermentation traits and increase fiber digestibility of tropical forages. Therefore, two *in vitro* experiments were conducted to evaluate the effects of *B. licheniformis* 809 and *B. subtilis* 810 in comparison with monensin, narasin, and flavomycin on ruminal fermentation parameters, gas production kinetics, and digestibility using two forage-based dietary scenarios.

## Material and methods

### Experimental location and ethical approval

These *in vitro* studies were conducted at the Beef Cattle Sector and Animal Nutrition Laboratory—LANA (Universidade Federal de Mato Grosso—UFMT; Cuiabá, MT, Brazil, −15°36′ 31.32″S; −56°03′49.32″W and 193 m elevation). The experiments were carried in accordance with the ethical principles adopted by the National Council for Animal Experimentation (CONCEA) and the experimental protocol was approved by the Ethics Committee in Animal Use (Comitê de Ética no Uso de Animais—CEUA/UFMT; approval n°23108.035716/2023).

### Experimental designs, treatments, and substrates

Two *in vitro* experiments were conducted to evaluate the effects of a Bacillus-based DFM (Bovacillus, Novonesis, Lyngby, Denmark), monensin (Mon; ionophore—Rumensin 100, Elanco Animal Health, São Paulo, SP, Brazil), Narasin (Nar; ionophore—Zimprova; Elanco Animal Health, São Paulo, SP, Brazil), and Flavomycin (Flavo; non-ionophore—Flavomycin 80; Huvepharma do Brazil, Porto Alegre, RS, Brazil) on ruminal fermentation parameters of forage-based diets. The DFM consisted of a mixture of *B. licheniformis* 809 and *B. subtilis* 810 (3.2 × 10^9^ CFU per g) and was evaluated at the manufacturer’s recommended dosage for beef cattle (2 g/head per day; **DFM1x**) and a 5X dose (10 g/head per day; DFM5x). The 5X dose was defined following recommendations by Silva et al. (2024), due to some specific additives requiring higher doses to be effective for *in vitro* fermentation studies.

The forage samples (*Urochloa brizantha* cv. Marandu) used as substrates were obtained at the experimental farm of the Federal University of Mato Grosso located at 15°47′11″S and 56°04′17″W, at an altitude 140 m, with a mean rainfall of 1267 mm and mean temperature of 26.1° C. The climate according to Köppen classification is Aw, tropical savanna, with the dry season in winter and the rainy season in the summer (Peel et al. 2007). The medium-quality forage (MF) was harvested via manual grazing simulation (De Vries 1995) during the rainy season (January and February; Exp.1), whereas the low-quality forage (LF) was harvested during the dry season (August and September; Exp. 2). The forage samples were collected a single time in each season in several points on an area of 12 ha. These pastures were established in 2007 with monoculture of Marandu grass in petroferric eutrophic, typical soil of the Cerrado Biome and have been used under continuous stocking without any specific management of fertilization. The forage samples were dried in an oven (65 °C/72 h) and were ground through a 1- mm screen using a Wiley mill (Model 4, Thomas-Wiley Laboratory Mill, Thomas Scientific, Swedesboro, NJ). Both forages were evaluated for their nutritional profile, including dry matter (DM), organic matter (OM), CP, ether extract (EE), neutral detergent fiber (NDF) and acid detergent fiber (ADF) contents, and the results are presented in Table 1.

**Table 1.** Ingredients percentage composition and chemical composition of the experimental diets (experiment 1 and 2).

**Item** ^a^	Exp. 1 (Medium quality forage—MF)		Exp. 2 (Low quality forage—LF)	
	MF	MF-S	LF	LF-S
***Composition, %DM***				
** Forage -Marandu grass**	100	88.17	100	84.35
** Ground flint corn**	-	10.58		12.68
** Soybean meal**	-	0.91	-	1.91
** Urea**	-	0.34	-	1.06
***Nutritional profile, %DM***				
** DM**	27.50	34.65	59.60	64.10
** CP**	9.64	10.82	3.00	7.55
** EE**	1.74	1.47	0.81	1.23
** Ash**	7.67	6.98	6.14	5.51
** NDF**	63.96	57.77	77.54	67.29
** ADF**	33.99	30.46	41.57	35.78

a MF, medium forage quality; MF-S, medium forage quality plus protein-energetic supplement; LF, low forage quality; LF-S, low forage quality plus protein-energetic supplement; DM, dry matter; CP, crude protein; EE, ethereal extract; NDF, Neutral detergent fiber; ADF, Acid detergent fiber.

In Exp. 1, the experimental treatments were arranged in a 2 × 6 factorial, having two substrates [Forage alone (MF) or combined with an energy-protein supplement (EPS; Table 1) - MF-S] as the factor 1, and six feed additive treatments [Control (Con), DFM1x, DFM5x, Mon, Nar, and Flavo] as the factor 2, resulting in 12 treatment combinations, as follows: (i) MF without feed additives inclusion, (ii) MF with DFM1x, (iii) MF with DFM5x, (iv) MF with inclusion of 20 ppm of Mon, (v) MF with inclusion of 13 ppm of Nar, (vi) MF with inclusion of 4 ppm of Flavo, (vii) MF-S without feed additives inclusion, (viii) MF-S with DFM1x, (ix) MF-S with DFM5x, (x) MF-S with 20 ppm of Mon, (xi) MF-S with 13 ppm of Nar, (xii) MF-S with 4 ppm of Flavo.

Similarly, in Exp. 2, LF and LF combined with EPS (LF-S) were paired with the same feed additive treatments as in Exp. 1. However, the nutritional profile of the EPS substrate was changed to meet the nutritional requirements of cattle grazing during the dry season (8% of CP in the diet), following recommendations of Detmann et al. (2014; Table 1).

The forage to EPS ratio of the substrates was 88.17:11.83 for MF-S and 84.35:15.65 for LF-S. These ratios were defined considering dry matter intake estimated following BR-Corte (2024) recommendations for a Nellore young bull weighing 300 kg on pasture and receiving supplement at a level of 0.3% of BW in the rainy and dry seasons.

The additive dosages were calculated to simulate conditions in the rumen of a 300-kg BW growing steer, assuming a rumen volume of 54 L (Church 1974). The EPS used in Exp. 1 was formulated to meet the nutrient requirements of a growing beef steer gaining approximately 0.800 kg/day, whereas in Exp. 2, the estimated daily gain was in the order of 0.350 kg (BR-Corte 2024).

The experimental units herein were the fermentation batches and eleven bottles per treatment were used in each run. In addition, 4 bottles were used as blanks (only rumen fluid, macro and micromineral, and a buffer solution) at 24, 48, and 96 h of incubation. For volatile fatty acids (VFA) analysis, the residual liquid of the three bottles for each treatment was collected at the 24-h time point. The residual material filtered at 24, 48, and 96 h was used to quantify *in vitro* digestibility of dry matter (IVDMD), organic matter (IVOMD), neutral detergent fiber (IVNDFD), acid detergent fiber (IVADFD), ruminal pH, and ruminal ammonia (NH_3_-N), with each treatment ­utilizing a total of 11 bottles.

For each bottle 125 mL, 0.5 g of the pre-dried substrate was weighed, being 0.5 g of forage for MF, 0.441 g of forage and 0.059 of EPS for MF-S, 0.5 g of forage for LF, and 0.422 g of forage and 0.078 g of EPS for LF-S. To minimize variation in the final additive concentration, the additives were pre-diluted 20 min before the incubations. Additives DFM1x, DFM5x, and Flavo were diluted in distiller water, whereas Mon and Nar were diluted in ethanol (adapted from Nagaraja et al. 1987). Subsequently, 0.2 mL of the additive-diluent solution was added to each bottle containing McDougall’s buffer (­McDougall 1948), delivering the pre-established concentration of each treatment. McDougall’s buffer (9.8 g/L of NaHCO_3_, 3.72 g/L of Na2HPO_4_, 0.57 g/L of KCl, 0.47 g/L of NaCl, 0.053 g/L of CaCl2.2H_2_O and 0.12 g/L of MgSO_4_.7H_2_O) was maintained in a water bath at 39 °C and continuously purged with CO_2_ for 40 min. Subsequently, resazurin (1.25 mL of 0.1% resazurin per 1 L of buffer) and a reducing agent (0.313 g L-cysteine-HCl plus 0.08 g of NaOH per L of buffer) were added to the buffer, homogenized, and incubated for 10 minutes at 39 °C under continuous CO_2_ purge. Resazurin was used as pH indicator, with a color change from purple to pink or colorless indicating appropriate anaerobic conditions (Tedeschi et al. 2009).

### Donor animals and ruminal fluid collection

Three rumen-fistulated, castrated Nellore × Angus steers (843 ± 50 kg BW) were used as donors of fresh rumen fluid. Prior to the first incubation, the steers were adapted for 14 d and remained on the same management regimen throughout the incubation period (four replicates; *n* = 4 per experiment). Animals were maintained on pasture with *Urochloa brizantha* cv. Marandu and supplemented daily at 08:00 AM with EPS at 0.3% of BW (CP = 20%; TDN = 65%). The diet did not contain any feed additives. Fresh rumen fluid was collected manually from the cranial, ventral, and caudal part of the rumen and filtered through four layers of cheesecloth directly into thermo bottles (one per steer). After collection, the ruminal fluid was immediately transported to the laboratory. The inoculum collected from each steer was then pooled, filtered through a Buchner funnel under CO_2_ purge, maintained in a water bath at 39 °C, and continuously purged with carbon dioxide (CO_2_) throughout the incubation.

Each bottle was filled with the buffer solution (40 mL) and rumen fluid (10 mL), adding up to a 1:4 rumen fluid: buffer ratio (vol/vol). Bottles were then continuously purged with CO_2_ for 10 seconds and immediately closed with rubber stoppers, crimped with aluminum seals. The pressure within each bottle was returned to zero by puncturing with a 21-gauge needle, and bottles were then maintained at 39°C in a shaking water bath (Dubnoff Agi. Orbital SL-158 Solab, Piracicaba, SP, Brazil).

### In vitro gas production

In each run (*n* = 4), incubations were conducted for 96 h, and GP t was recorded at 3, 6, 12, 24, 36, 48, 72, and 96 h from the start of incubation by semiautomatic pressure transducers (Data Logger GN200, MPL, Piracicaba, SP, Brazil), following the technique described by Theodorou et al., (1994) and ­Mauricio et al., (1999). Final total gas production (**TGP**) volume was corrected for inoculum contribution by subtracting the mean TGP of the blank bottles. The resulting volume was then expressed as mL/g of DM using the following equation:


$$\text{Exp}\;.\;1:\;\mathrm{Gas}\;\mathrm{volume}\;(mL/g\;DM)=(5.4845\times \mathrm{Gas}\;\mathrm{pressure}(\mathrm{psi}))-0.2353;\;r^{2}=0.9998$$

$$\text{Exp}\;.\;2:\;\mathrm{Gas}\;\mathrm{volume}\;(mL/g\;DM)=(5.4749\times \mathrm{Gas}\;\mathrm{pressure}(\mathrm{psi}))-0.0275;\;r^{2}=0.9992$$

For TGP over time, the cumulative pressure values were adjusted according to Schofield et al. (1994):


$$\text{Exp}\;.\;1:\;\text{Vt}=[V1/(1+e^{2+4\times [K1\times (L-\text{Time})]})]+[V2/(1+e^{2+4\times [K2\times (L-\text{Time})]})]$$

$$\text{Exp}\;.\;\;2:\;\text{Vt}=[V1/(1+e^{2+4\times [K1\times (L-\text{Time})]})]\;$$

where: Vt = gas volume produced up to the specific time, mL; V1 and V2 = maximum gas volume achieved from complete digestion of each pool, mL; K1 and K2 = specific rate of digestion of each pool, h-1; L = lag time, h.

### Digestibility of DM, OM, NDF, and ADF

Of the 11 bottles incubated per treatment per run, 3 were removed at 24, 48 and 96 h of incubation to evaluate IVDMD, IVOMD, IVNDFD, and IVADFD. Upon removal, each bottle was immediately placed in ice-cold water to halt the fermentation. The incubated material (substrate/rumen fluid/buffer) was filtered through Ankom^®^ F57 bags (Ankom Technology Corp., Macedon, NY) and oven-dried at 105°C for 24 h. Subsequently, the bags were weighed and sealed for sequential determination of NDF and ADF, according to Van Soest et al. (1991). Nutrient digestibility was calculated based on the difference in residue weight after incubation.

The IVOMD was calculated using the following equations proposed by Menke and Steingass (1988):


IVOMD, %=16.49+0.9042×GP+0.0492×CP+0.0387×TA,

where GP: gas production, CP: crude protein, TA: total ash. GP is expressed in mL/g; CP, and TA as g/kg- DM.

### Rumen ammonia-N concentration and microbial biomass yield

Approximately 15 mL of the ruminal fluid were collected, pooled across all sampling times (24, 48, and 96 h), measured for the pH, and stored at −20°C for subsequent analysis of NH_3_-N concentration. The samples were thawed and centrifuged (15000 × *g*), whereas NH_3_-N was determined according to procedures described by Chaney and Marbach (1962). Absorbance was read at 630 nm using the Gen5 Microplate Reader and Imager Software (BioTek Instruments, version 1.10.8, Winooski, VT, USA). The microbial biomass yield (MBY) was estimated from Blümmel et al. (1997):


MBY=IVDMD (mg)−(GP×2.2 mg/mL),

Where GP is gas production (mL/g of DM) in 24 h of incubation.

### Volatile fatty acids

For VFA analysis, 2 mL subsamples of the rumen/buffer mixture were collected prior to incubation and from each bottle at 24 h. Samples were filtered through Ankom^®^ F57 bags, centrifuged at 1000 × *g* for 3 min, and the supernatant was transferred to clean microcentrifuge tubes. These were centrifuged again at 12,000 × *g* for 10 min at 4 °C. The clarified fluid was then transferred to a vial containing formic acid (100 µL/mL) for preservation. The VFA concentrations were determined using gas chromatograph (Agilent 7890A, Agilent Technologies, USA) equipped with an automatic injector and a flame ionization detector (**FID**). Separation was achieved using DB-WaxETR capillary column (30 m, 0.25 mm, 0.25 µm) was used. The carrier gases used were synthetic air, N, and H. Chromatographic conditions were: injector temperature of 250°C; oven temperature starting at 120°C with a ramp up to 200°C, and FID maintained at 300°C. Elution times and peak areas were analyzed using OpenLab software (Agilent Technologies, USA). The VFA concentrations were quantified using at six six concentrations for each compound: acetic acid (695092), propionic acid (402907), butyric acid (B103500), isobutyric acid (I-1754), isovaleric acid (129542), and valeric acid (240370), all from Sigma- (Merck KGaA, Darmstadt, Germany).

### Statistical analysis

All statistical analyses were performed using SAS (version 9.4; SAS Institute Inc., Cary, NC). Residual normality and variance homogeneity were determined using the UNIVARIATE procedure of SAS. For Exp. 1 and 2, data were analyzed in a completely randomized design considering a 2 × 6 factorial arrangement, as previously explained and described. The average values obtained from the bottles within each incubation were considered as the experimental unit for all analyses reported herein (Udén et al. 2012). The fitting of the data was performed using the GLIMMIX procedure of SAS, considering substrates, additives, and the interaction between substrates and additives as fixed effects and run (incubation) as the random effect (Christodoulou et al. 2025). When a significant interaction was identified, the additive effects were evaluated within each substrate. When the insignificant interaction was identified, the main factors (substrates and additives) were evaluated and reported separately. The dynamics of *in vitro* gas production over time were estimated using NLIN option of SAS. The parameters of the nonlinear functions as well as all other variables were fitted through a generalized mixed model and compared by using Fisher’s least significant difference (LSD). The DFM dosages (1× and 5×) were also analyzed for linear and quadratic responses. As DFM doses were non-equidistant, orthogonal contrasts were obtained using the interactive matrix language (IML procedure). For all the analyses, differences detected at *P* ≤ 0.05 were considered significant and tendencies were denoted if 0.05 < *P* ≤ 0.10.

## Results

### Experiment 1: Medium-quality forage

#### Gas production, kinetic parameters, and digestibility

There were no significant interactions between substrates and feed additives (*P *> 0.05) for TGP, kinetic parameters (Table 2), and nutrient digestibility (Table 3). Therefore, the results were presented based on the main effects only.

**Table 2.** *In Vitro* gas production (mL/g substrate) and kinetics parameter estimates of high-quality forage-based diets with different additives (exp. 1).

Item^a^	Gas production, mL/g substrate				kinetic variables^b^				
	12h	24 h	48 h	96 h	V1	K1	L	V2	K2
**Substrates**									
** MF**	84.30	150.02	227.02	267.40	87.26	0.097	5.70	175.83	0.0202
** MF-S**	94.25	166.26	244.02	280.86	100.76	0.099	5.48	175.75	0.0211
**SEM**	1.586	1.320	1.744	1.557	1.623	0.001	0.211	1.715	0.001
**Additives**									
** Con**	91.14^b^	159.71^b^	237.38^a^	270.95^b^	92.67^a^ ^b^	0.1006^a^ ^b^	5.59^b^ ^c^	174.18^b^	0.0215^a^
** DFM1x**	95.88^a^ ^b^	164.98^a^	241.29^a^	274.16^b^	97.16^a^	0.0980^b^	5.22^b^ ^c^ ^d^	173.04^b^	0.0215^a^
** DFM5x**	96.64^a^	165.98^a^	241.60^a^	273.64^b^	97.55^a^	0.0972^b^	5.13^c^ ^d^	171.56^b^	0.0216^a^
** Mon**	77.93^c^	144.64^d^	217.64^b^	260.74^c^	89.88^b^	0.0921^c^	6.06^a^ ^b^	165.94^c^	0.0194^b^
** Nar**	80.35^c^	150.90^c^	237.29^a^	294.66^a^	89.83^b^	0.1038^a^	6.88^a^	199.96^a^	0.0186^c^
** Flavo**	93.72^a^ ^b^	162.63^a^ ^b^	237.93^a^	270.64^b^	96.95^a^	0.0972^b^	4.66^d^	170.05^b^ ^c^	0.0212^a^
**SEM**	2.110	1.900	2.174	2.122	2.261	0.002	0.336	2.324	0.001
***P-value***									
** Substrate**	<.0001	<.0001	<.0001	<.0001	<.0001	0.1358	0.4029	0.9579	<.0001
** Additive**	<.0001	<.0001	<.0001	<.0001	0.0100	0.0007	0.0004	<.0001	<.0001
** Subs × Add^d^**	0.8541	0.7668	0.2836	0.2892	0.8652	0.6950	0.8209	0.1801	0.8642
***DFM orthogonal effect***									
** Linear**	0.0745	0.012	0.069	0.289	0.1679	0.2146	0.4108	0.3580	0.6723
** Quadratic**	0.1093	0.306	0.360	0.395	0.1694	0.3736	0.5081	0.8064	0.7636

a MF—medium forage quality; MF-S—medium forage quality plus protein-energetic supplement; (Con): diet control without additive; Bovacillus 1x (DFM1x); Bovacillus 5x (DFM5x); Monensin (Mon); Narasina (Nar) and Flavomycin (Flavo); SEM—Standard error of mean; Subst x add: effects ot the substrate and additive.

b V1 and V2= Maximum gas volume of each pool (mL); K1 = Specific rate of digestion (h^−1^); L = lag time (h).

c Least square means with different superscripts on the same column differ at *P *≤ 0.05.

d Substrate × additive interaction.

**Table 3.** *In Vitro* of dry matter digestibility (IVDMD), organic matter digestibility (IVOMD), neutral detergent insoluble fiber digestibility (IVNDFD), and acid detergent insoluble fiber digestibility (IVADFD) of high-quality forage-based diets with different additives (exp. 1).

**Item** ^a^	IVDMD, %			IVOMD, %^b^			IVNDFD, %			IVADFD, %		
	24 h	48 h	96 h	24 h	48 h	96 h	24 h	48 h	96 h	24 h	48 h	96 h
**Substrates**												
** MF**	62.70	72.63	79.98	51.33	65.26	72.56	50.58	67.54	75.88	52.07	67.49	75.37
** MF-S**	64.91	74.54	81.80	54.58	68.65	75.31	51.89	68.63	77.05	53.89	68.75	76.15
**SEM**	0.824	1.178	0.862	0.289	0.316	0.282	0.389	0.649	1.033	0.762	1.291	1.821
**Additives**												
** Con**	64.83^a^	74.84^a^	82.72^a^	53.24^b^	67.29^a^	73.36^b^	52.78^a^	70.40^a^ ^b^	78.37^a^	55.64^a^	70.59^a^	77.00^a^
** DFM1x**	64.66^a^	74.98^a^	82.81^a^	54.19^a^	68.00^a^	73.94^b^	52.65^a^	70.17^a^ ^b^	78.65^a^	54.35^a^	70.17^a^	77.90^a^
** DFM5x**	64.06^a^ ^b^	75.05^a^	82.23^a^ ^b^	54.37^a^	68.05^a^	73.85^b^	52.76^a^	70.71^a^	78.56^a^	55.21^a^	70.39^a^	77.62^a^
** Mon**	63.19^b^	71.97^b^ ^c^	77.70^d^	50.52^d^	63.72^b^	71.51^c^	48.83^b^	64.09^c^	72.12^d^	49.65^b^	64.32^b^	72.84^c^
** Nar**	61.44^c^	71.33^c^	79.42^c^ ^d^	51.65^c^	67.27^a^	77.65^a^	47.60^b^	64.28^c^	74.57^c^	48.91^b^	64.21^b^	73.46^b^ ^c^
** Flavo**	64.65^a^	73.32^a^ ^b^	80.46^b^ ^c^	53.77^a^ ^b^	67.39^a^	73.30^b^	52.80^a^	68.88^b^	76.54^b^	54.12^a^	69.05^a^	75.75^a^ ^b^
**SEM**	0.887	1.280	1.011	0.344	0.393	0.384	0.606	0.786	1.113	0.905	1.384	1.966
***P-value***												
** Substrate**	<.001	<.001	0.002	<.001	<.001	<.001	0.008	0.019	0.008	<.001	0.017	0.2996
** Additive**	<.001	<.001	<.001	<.001	<.001	<.001	<.001	<.001	<.001	<.001	<.001	<.001
** Subs × Add^d^**	0.160	0.931	0.204	0.765	0.282	0.289	0.548	0.797	0.270	0.933	0.853	0.662
***DFM orthogonal effect***												
** Linear**	0.1602	0.8345	0.5273	0.0377	0.1508	0.4637	0.9735	0.5697	0.8769	0.9951	0.9364	0.7789
** Quadratic**	0.9742	0.9000	0.8267	0.0729	0.1440	0.2525	0.8640	0.6816	0.7143	0.1303	0.6344	0.5154

a MF—medium forage quality; MF-S—medium forage quality plus protein-energetic supplement; (Con): diet control without additive; Bovacillus 1x (DFM1x); Bovacillus 5x (DFM5x); Monensin (Mon); Narasina (Nar) and Flavomycin (Flavo); SEM—Standard error of mean; Subst x add: effects ot the substrate and additive.

b The *in vitro* organic matter was calculated according to Menke and Steingass (1988): IVOMD%= 16.49 + 0.9042GP + 0.0492CP + 0.0387TA.

c Least square means with different superscripts on the same column differ at *P *≤ 0.05.

d Substrate × additive interaction.

The TGP at 12, 24, 48, and 96 h was greater for MF-S compared with MF (*P* < 0.01; Table 2). Additionally, the TGP on the first pool (V1) and rates of digestion for the second pool (K2) were higher (*P* < 0.01) for MF-S compared with MF. The effects of feed additives on TGP in all times of evaluation were significant (*P* < 0.05; Table 2). The inclusion of DFM5x increased (*P* < 0.01) the TGP at the early timepoints of incubation (12 and 24 h), whereas Mon and Nar decreased TGP compared with the Con (*P* < 0.01; Table 2). At 48 h of incubation, Nar presented intermediate values, and did not differ (*P* < 0.01) from the Con, DFM1x, DFM5x, or Flavo, while Mon reduced TGP compared to Con and all other feed additives (*P* < 0.01; Table 2). At 96 h, Nar increased TGP (*P* < 0.01) compared with Con and other treatments, whereas Mon decreased TGP (*P* < 0.01). The DFM1x, DFM5x, and Flavo did not differ from Con as this timepoint (*P* > 0.05). Additionally, DFM inclusion resulted in a linear increase (*P* < 0.012) in TGP at 24 h (Table 2).

The inclusion of DFM (both 1x and 5x) did not modify (*P* *>* 0.05) the first TGP pool (V1) compared with Con and Flavo. Mon and Nar did not change (*P* < 0.01) V1 compared with Con but decrease V1 compared with DFM and Flavo (Table 2). Comparable results were observed for the second TGP pool (V2), except for Nar, which increased (*P* < 0.01) V2 compared with the other treatments. Specific rates of digestion for the first and second pools (K1 and K2) did not change (*P* > 0.05) with DFM inclusion when compared with Con and Flavo, but Mon decreased (*P* < 0.05) K1 and K2 compared to all treatments (Table 2). When compared with Con, Nar did not alter (*P* > 0.05) K1 but increased (*P* < 0.01) K1 compared with all other treatments (*P* < 0.01). However, similar to Mon, Nar also decreased K2 (*P* < 0.01). The lag time was higher (*P* = 0.0004) for ionophores (Mon and Nar) and lower (*P* < 0.01) for Flavo compared with Con, DFM1x, and DFM5x (Table 2).

For digestibility, MF-S had higher (*P* < 0.05) IVDMD, IVOMD, IVNDFD, and IVADFD at all the evaluated timepoints when compared with MF, except to IVADFD that did not differ at 96 h (*P* = 0.30; Table 3).

Feed additive effects were also detected (*P* *<* 0.05) on *in vitro* nutrient digestibility (Table 3). Regardless of the dosage, DFM and Flavo inclusion did not modify (*P* > 0.05) IVDMD, IVOMD, IVNDFD, and IVADFD compared with Con; the exception being the lower IVDMD and IVNDFD for Flavo vs. CON at 96 h (*P* < 0.01). Overall, Mon and Nar reduced (*P* < 0.01) IVDMD, IVOMD, IVNDFD, and IVADFD compared with Con, DFM1x (*P* < 0.01), except for IVOMD at 48 and 96 h, that Nar did not differ (*P* > 0.05) from the Con (Table 3).

#### NH_3_-N, pH, and microbial biomass yield

There was significant interaction between substrates and feed additives (*P* < 0.05) for NH_3_-N concentrations at 24, 48, and 96 h (Table 4). At 24 h of fermentation using MF, Mon increased NH_3_-N compared with all other treatments (*P* = 0.02) but did not differ from Flavo (*P* > 0.05). When substrate was MF-S, DFM5x, Mon, and Flavo did not differ from Con (*P* > 0.05), whereas DFM1x and Nar reduced NH_3_-N concentrations (*P* = 0.02). At 48 h, with MF as the substrate, DFM1x did not differ (*P* > 0.05) from Con, while DFM5x, Mon, Nar, and Flavo reduced (*P* < 0.01) NH_3_-N concentrations compared with Con. When substrate was MF-S, NH_3_-N at 48 h was higher (*P* < 0.01) for DFM1x and Flavo compared with Con; DFM5x did not differ (*P* > 0.05) from Con, whereas Mon and Nar reduced NH_3_-N compared with other treatments (*P* < 0.01). At 96 h, with MF, Flavo resulted in the highest NH_3_-N concentrations (*P* < 0.01); DFM5x and Nar showed greater values than Con and DFM1x (*P* < 0.01), whereas Mon resulted in the lowest NH_3_-N concentrations (*P* < 0.01). When MF-S was used, DFM5x increased NH_3_-N compared with all other treatments (*P* < 0.01), while DFM1x, Mon, Nar, and Flavo decreased NH_3_-N compared with Con (*P* < 0.01; Table 4).

**Table 4.** *In Vitro* ruminal ammonia-N (NH_3_-N), pH, and microbial biomass yield (MBY) of high-quality forage-based diets with different additives (exp. 1).

**Substrates** ^a^	Additives^b^	NH_3_-N, mg/dL			pH			MBY, mg/kg
		24 hours	48 hours	96 hours	24 hours	48 hours	96 hours	
**MF**	Con	2.30^b^ ^c^	8.40^a^	9.71^c^	6.74	6.60	6.69	483.29
	DFM1x	2.07^c^	8.77^a^	9.30^c^	6.67	6.61	6.71	477.60
	DFM5x	1.86^c^	6.08^c^	10.90^b^	6.62	6.55	6.70	455.58
	Mon	3.02^a^	4.95^d^	6.49^d^	6.66	6.57	6.61	423.55
	Nar	1.93^c^	5.71 ^c^ ^d^	10.79^b^	6.71	6.58	6.55	466.91
	Flavo	2.70^a^ ^b^	7.16^b^	12.57^a^	6.68	6.51	6.67	469.10
**MF-S**	Con	3.38^a^	10.76^c^	16.71^b^	6.64	6.62	6.70	463.65
	DFM1x	2.56^c^	11.69^a^ ^b^	14.92^c^	6.72	6.63	6.71	450.14
	DFM5x	3.15^a^ ^b^	11.04^b^ ^c^	18.58^a^	6.69	6.61	6.67	452.04
	Mon	3.51^a^	6.66^e^	10.40^e^	6.65	6.59	6.59	446.15
	Nar	2.92^b^ ^c^	9.66^d^	12.58^d^	6.68	6.57	6.53	448.50
	Flavo	3.11^a^ ^b^	11.93^a^	15.05^c^	6.71	6.64	6.67	416.42
**SEM^c^**		0.198	0.390	0.338	0.085	0.071	0.033	13.882
***Substrates***								
** MF**	-	2.31	6.84	9.96	6.68	6.57	6.65	462.67
** MF-S**		3.10	10.29	14.70	6.68	6.61	6.64	446.15
***Additives***								
** Con**	-	2.84	9.58	13.21	6.69	6.61	6.69^a^	473.47
** DFM1x**	-	2.31	10.23	12.11	6.69	6.62	6.71^a^	463.87
** DFM5x**	-	2.50	8.56	14.74	6.65	6.58	6.68^a^	453.81
** Mon**	-	3.26	5.81	8.44	6.65	6.58	6.60^b^ ^c^	434.85
** Nar**	-	2.43	7.68	11.68	6.70	6.57	6.54^c^	457.71
** Flavo**	-	2.90	9.54	13.81	6.70	6.58	6.67^a^ ^b^	442.76
***P-value***								
** Substrate**	-	<.0001	<.0001	<.0001	0.9336	0.1239	0.6417	0.0472
** Additive**	-	<.0001	<.0001	<.0001	0.8618	0.8140	0.0003	0.0987
** Subs × Add^d^**		0.0153	<.0001	<.0001	0.5538	0.6807	0.9807	0.1797
***DFM orthogonal effect***								
** Linear**		0.2178	<.0001	<.0001	0.4056	0.3393	0.5577	0.1880
** Quadratic**		0.0016	0.0020	<.0001	0.7771	0.6388	0.5726	0.6590

a MF—medium forage quality; MF-S—medium forage quality plus protein-energetic supplement.

b Con: diet control without additive; Bovacillus 1x (DFM1x); Bovacillus 5x (DFM5x); Monensin (Mon); Narasina (Nar) and Flavomycin (Flavo).

c Least square means with different superscripts on the same column differ at *P *≤ 0.05.

d Substrate × additive interaction.

e Explanatory note.

There was no significant interaction (*P* > 0.05) between substrates and feed additives (Table 4) for *in vitro* rumen pH. Additive effects on pH were detected only at 96 h (*P* < 0.01), with Mon and Nar reducing the pH compared to Con, DFM1x, DFM5x, and Flavo (Table 4). However, Flavo did not differ (*P* > 0.05) of the Con, DFM1x, DFM5x and Mon. For microbial biomass yield there was a significant effect (*P* = 0.047) of substrate only, with higher values observed for MF compared with MF-S (Table 4).

#### VFA

There was a significant interaction between substrate × feed additive (*P* = 0.03) for propionate only (Table 5). Substrate effects (*P* < 0.01) were observed for ruminal molar proportions of acetate and butyrate (Table 5), with acetate being higher and butyrate being lower for MF compared with MF-S (*P* < 0.05).

**Table 5.** *In Vitro* total volatile fatty acids (VFA) and VFA profile of high-quality forage-based diets with different additives (exp. 1).

**Substrates** ^a^	Additives^b^	total VFA	Volatile fatty acids, mmol/100 mmol^c^						
			Acet	Prop	But	Isobut	Val	Isoval	Acet: prop
**MF**	Con	36.45	62.47	19.69^c^	9.16	2.93	2.71	3.04	3.18
	DFM1x	37.87	63.42	18.92^c^	9.02	2.93	2.68	3.03	3.36
	DFM5x	38.72	63.27	19.50^c^	8.83	2.82	2.62	2.96	3.25
	Mon	40.05	61.16	23.04^a^	7.81	2.67	2.53	2.79	2.66
	Nar	40.34	62.48	21.44^b^	8.04	2.69	2.56	2.80	2.92
	Flavo	39.57	63.82	19.37^c^	8.51	2.76	2.62	2.94	3.30
**MF-S**	Con	40.62	63.04	19.10^c^	9.56	2.76	2.60	2.94	3.30
	DFM1x	38.93	62.93	19.03^c^	9.54	2.83	2.67	3.01	3.32
	DFM5x	37.26	62.50	19.07^c^	9.63	2.92	2.77	3.12	3.29
	Mon	40.98	59.03	24.64^a^	8.34	2.65	2.55	2.80	2.41
	Nar	40.28	62.11	21.42^b^	8.36	2.69	2.61	2.82	2.91
	Flavo	38.68	62.56	19.87^c^	9.03	2.82	2.69	3.03	3.15
**SEM^e^**	-	2.110	0.425	0.499	0.122	0.136	0.130	0.150	0.082
***Substrates***									
** MF**		38.83	62.77	20.33	8.56	2.80	2.62	2.93	3.11
** MF-S**		39.46	62.03	20.52	9.07	2.78	2.65	2.95	3.06
***Additives***									
** Con**	-	38.54	62.76^a^ ^b^	19.39	9.36^a^	2.85^a^	2.66	2.99^a^	3.24^a^
** DFM1x**	-	38.40	63.18^a^	18.98	9.28^a^	2.88^a^	2.67	3.02^a^	3.34^a^
** DFM5x**	-	37.99	62.88^a^ ^b^	19.28	9.23^a^	2.87^b^	2.70	3.04^a^	3.27^a^
** Mon**	-	40.51	60.09^c^	23.84	8.07^c^	2.66^b^	2.54	2.80^b^	2.53^c^
** Nar**	-	40.31	62.29^b^	21.43	8.20^c^	2.69^b^	2.58	2.81^b^	2.91^b^
** Flavo**	-	39.13	63.19^a^	19.62	8.77^b^	2.79^a^	2.65	2.98^a^	3.23^a^
***P-value***									
** Substrate**	-	0.3948	0.0041	0.3096	<.0001	0.5847	0.4804	0.4799	0.1999
** Additive**	-	0.2512	<.0001	<.0001	<.0001	0.0061	0.2175	0.0008	<.0001
** Subs × Add^c^**		0.3009	0.0597	0.0289	0.3963	0.3831	0.5801	0.4737	0.0507
***DFM orthogonal effect***									
** Linear**		0.6528	0.9712	0.9193	0.3112	0.804	0.5643	0.4788	0.9061
** Quadratic**		0.9807	0.3054	0.1969	0.6221	0.6504	0.9012	0.7695	0.1137

a MF—medium forage quality; MF-S—medium forage quality plus protein-energetic supplement.

b Acet: acetate, Prop: Propionate, But: Butyrate, Isobut: Iso-butyrate, Val: Valerate, Isoval: Iso-valerate, Acet: prop: Acetate: Propionate.

c Con: diet control without additive; Bovacillus 1x (DFM1x); Bovacillus 5x (DFM5x); Monensin (Mon); Narasina (Nar) and Flavomycin (Flavo).

d Least square means with different superscripts on the same column differ at *P *≤ 0.05.

e Substrate × additive interaction.

No effects of substrate or additives effects (*P* > 0.05) were detected on total VFA concentrations. The inclusion of DFM1x, DFM5x, Nar, and Flavo did not modify (*P* > 0.05) acetate concentrations compared with Con, but Mon reduced it (Table 5). Moreover, Mon increased propionate concentration compared with all other treatments (*P* = 0.03); Nar had intermediate values, whereas DFM1x, DFM5x and Flavo did not differ (*P* > 0.05) from Con or from each other (Table 5). The inclusion of DFM1x and DFM5x did not modify (*P* > 0.05) butyrate concentration compared with Con; Flavo had intermediate value, whereas Mon and Nar resulted in lower concentrations (*P* < 0.01). For isobutyrate, DFM1x and Flavo did not differ (*P* > 0.05) from Con, whereas DFM5x, Mon, and Nar reduced isobutyrate compared with other treatments (*P* < 0.01). Isovalerate showed a similar pattern to isobutyrate, except that DFM5x did not differ (*P* > 0.05) from Con, DFM1x and Flavo. Acetate-to-propionate ratio was similar (*P* < 0.01) among Con, DFM1x, DFM5x, and Flavo; an intermediate ratio was observed for Nar, and a lower ratio was reported for Mon (*P* < 0.01; Table 5).

### Experiment 2: Low-quality forage

#### Gas production, kinetic parameters, and digestibility

No significant interaction between substrate and feed additive was identified for TGP at 12 h of incubation (*P* = 0.33; Table 6). However, substrate × additive interactions were observed for TGP when measured at 24, 48, and 96 h of fermentation (*P* < 0.05; Table 6).

**Table 6.** *In Vitro* gas production (GP; mL/g substrate) and kinetics parameter estimates of low-quality forage-based diets with different additives (exp. 2).

**Substrates** ^a^	Additives^b^	Gas production, mL/g substrate				kinetics variables^c^		
		12h	24 h	48 h	96 h	V1	K1	L
**LF**	Con	34.55	80.70^a^	153.39^a^	209.63^a^	197.68^b^	0.02233	8.75
	DFM1x	37.76	83.63^a^	156.36^a^	215.92^a^	204.58^a^	0.02238	8.48
	DFM5x	35.27	81.31^a^	154.19^a^	211.60^a^	200.03^a^ ^b^	0.02273	8.88
	Mon	25.64	62.58^b^	132.51^b^	191.56^b^	183.33^c^	0.02093	10.40
	Nar	22.37	60.08^b^	126.48^c^	182.88^c^	173.70^d^	0.02190	11.03
	Flavo	34.73	81.09^a^	154.38^a^	212.68^a^	200.60^a^ ^b^	0.02235	8.79
**LF-S**	Con	49.83	112.18^a^ ^b^	184.85^a^ ^b^	235.84^a^	221.43^a^	0.02585	7.04
	DFM1x	51.56	114.53^a^	187.62^a^	235.67^a^	222.50^a^	0.02645	7.04
	DFM5x	49.17	111.72^a^ ^b^	185.53^a^ ^b^	233.33^a^ ^b^	218.95^a^	0.02653	7.23
	Mon	38.86	89.86^d^	164.30^d^	214.03^c^	202.40^c^	0.02458	8.40
	Nar	39.17	97.40^c^	174.43^c^	226.33^b^	212.40^b^	0.02555	8.57
	Flavo	47.14	110.34^b^	180.31^b^	232.52^a^ ^b^	217.60^a^ ^b^	0.02598	7.24
**SEM^e^**	-	1.296	1.981	2.648	6.025	5.473	0.001	0.262
***Substrates***								
** LF**	-	31.72	74.90	146.22	204.04	193.32	0.02210	9.39
** LF-S**		45.95	106.00	179.51	229.62	215.88	0.02582	7.59
***Additives***								
** Con**	-	42.19 ^b^	96.44	169.12	222.73	209.55	0.02409^a^ ^b^	7.89^b^
** DFM1x**	-	44.66^a^	99.08	171.99	225.79	213.54	0.02441^a^ ^b^	7.76^b^
** DFM5x**	-	42.22^b^	96.51	169.86	222.47	209.49	0.02463^a^	8.05^b^
** Mon**	-	32.25^c^	76.22	148.40	202.79	192.86	0.02275^c^	9.40^a^
** Nar**	-	30.77^c^	78.74	150.45	204.60	193.05	0.02373^b^	9.80^a^
** Flavo**	-	40.93 ^b^	95.72	167.35	222.60	209.10	0.02416^a^ ^b^	8.01^b^
***P-value***								
** Substrate**	-	<.0001	<.0001	<.0001	<.0001	<.0001	<.0001	<.0001
** Additive**	-	<.0001	<.0001	<.0001	<.0001	<.0001	0.0003	<.0001
** Subs × Add^e^**		0.3284	0.0331	<.0001	0.0003	0.0002	0.9822	0.3149
***DFM orthogonal effect***								
** Linear**		0.3819	0.5292	0.9005	0.5667	0.4813	0.1976	0.3287
** Quadratic**		0.0121	0.0543	0.1388	0.1873	0.0573	0.5258	0.4611

a LF-S—low forage quality plus protein-energetic supplement.

b Con: diet control without additive; Bovacillus 1x (DFM1x); Bovacillus 5x (DFM5x); Monensin (Mon); Narasin (Nar) and Flavomycin (Flavo).

c V1—Maximum gas volume of each pool, mL; K1—Specific rate of digestion, h^−1^; L—lag time, h.

d Least square means with different superscripts on the same column differ at *P *≤ 0.05.

e Substrate × additive interaction.

*In vitro* TGP was greater (*P* < 0.01) for LF-S compared with LF at 12 h of incubation. Moreover, *in vitro* TGP increased quadratically (*P* = 0.01) at 12 h of fermentation with inclusion of DFM. On the other hand, Mon and Nar reduced TGP at 12 h compared to the other treatments (*P* < 0.01), whereas Flavo did not alter TGP relative to Con (*P* > 0.05; Table 6).

When the substrate was LF, TGP was not affected (*P* > 0.05) by the inclusion of DFM (DFM1x and DFM5x) or Flavo compared with Con at 24, 48, and 96 h. In contrast, Mon and Nar resulted in lower TGP values at these timepoints (*P* < 0.01). A similar response was observed when the substrate was LF-S, except that Flavo yielded intermediate values (Table 6).

A significant substrate × feed additive interaction (*P* < 0.01) was observed for TGP on V1 (Table 6). When substrate was the LF, inclusion of DFM1x resulted in greater V1 compared with Con, Mon, and Nar (*P* < 0.01), but did not differ (*P* > 0.05) from DFM5x and Flavo. The Mon and Nar had lower V1 compared to all other treatments (*P* < 0.01), with Mon being greater than Nar (*P* < 0.01). When the substrate was LF-S, DFM1x, DFM5x, and Flavo did not modify V1 compared with Con (*P* > 0.05); Nar was intermediate (*P* *<* 0.01), and Mon had the lowest values (*P* *<* 0.01; Table 6).

The K1 was greater, and the lag time was shorter for LF-S compared with LF (*P* = 0.0001). The digestion rate did not differ (*P* > 0.05) among Con, DFM1x, DFM5x, and Flavo; Nar showed an intermediate value (*P* *<* 0.01), without a difference from Con, DFM1x and Flavo; while Mon showed a lower value (*P* *<* 0.01). Lag time was greater (*P* < 0.01) for Mon and Nar compared with the other treatments, which did not differ from each other (*P* > 0.05; Table 6).

There was significant substrate × feed additives interaction (*P* < 0.05; Table 7) for IVDMD (24 h), IVOMD (24, 48, and 96 h), IVNDFD (24 h), and IVADFD (24 and 96 h; Table 7). Supplementation with DFM1x and DFM5x did not affect (*P* > 0.05) IVDMD at 24 h compared with Con, regardless of the substrate; however, IVDMD was greater for DFM1x and DFM5x vs. than for the other feed additives (*P* < 0.05). Flavo showed intermediate values. Monensin yielded higher IVDMD than Nar when substrate was LF (*P* *<* 0.01), but no differences were detected between these two ionophores when substrate was LF-S (*P* > 0.05). Similarly, IVOMD at 24, 48, and 96 h did not differ (*P* > 0.05) among Con, DFM1x and DFM5x for either substrate. The lowest IVOMD values were observed for Mon and Nar (*P* < 0.05), with Mon being greater than Nar when LF was used (*P* < 0.05), and Nar exciding Mon when LF-S was the substrate (*P* < 0.05). For IVNDFD at 24 h, DFM1x and DFM5x did not differ from Con (*P* > 0.05) in either substrate, but all three were greater than Mon, Nar, and Flavo (*P* < 0.01). Flavo was higher than Mon and Nar, which did not differ from each other (*P* > 0.05). Similar patterns were observed for IVADFD at 24 and 96 h.

**Table 7.** *In Vitro* digestibility of dry matter (IVDMD), organic matter (IVOMD), neutral detergent insoluble fiber (IVNDFD), and acid detergent insoluble fiber (IVADFD) of low-quality forage-based diets with different additives (exp. 2).

**Substrates** ^a^	Additives^b^	IVDMD, %			IVOMD^c^, %			IVNDFD, %			IVADFD, %		
		24h	48h	96h	24h	48h	96h	24h	48h	96h	24h	48h	96h
**LF**	Con	29.83^a^	44.77	55.41	33.15^a^	46.30^a^	56.47^a^	20.24^a^	36.09	49.20	15.78^a^	28.86	40.50^a^
	DFM1x	30.53^a^	44.55	55.61	33.68^a^	46.84^a^	57.61^a^	19.74^a^	36.48	50.03	16.19^a^	29.16	41.51^a^
	DFM5x	29.87^a^	43.85	54.38	33.27^a^	46.45^a^	56.83^a^	19.38^a^	36.33	49.29	15.40^a^	28.91	41.20^a^
	Mon	25.65^c^	39.16	51.21	29.88^b^	42.52^b^	53.20^b^	12.50^c^	29.18	43.38	8.00^c^	19.65	36.48^b^
	Nar	23.96^d^	40.42	50.07	29.43^b^	41.43^c^	51.63^c^ ^d^	11.24^c^	30.66	41.93	6.58^c^	21.84	34.61^b^
	Flavo	27.76^b^	39.22	55.21	33.22^a^	46.48^a^	57.02^a^	15.72^b^	31.08	49.24	12.48^b^	25.02	42.66^a^
**LF-S**	Con	43.13^a^	54.79	62.61	41.03^a^	54.17^a^ ^b^	63.39^a^	27.26^a^	41.26	52.28	23.76^a^	35.26	46.11^a^
	DFM1x	42.59^a^	54.51	62.37	41.45^a^	54.67^a^ ^d^	63.36^a^	26.84^a^	41.30	53.11	24.63^a^	35.04	45.53^a^
	DFM5x	42.47^a^	53.53	62.20	40.94^a^	54.29^a^ ^b^	62.94^a^ ^b^	27.40^a^	41.34	52.23	24.69^a^	34.56	45.18^a^
	Mon	35.53^c^	46.96	57.89	36.99^c^	50.45^d^	59.44^c^	15.97^c^	30.53	43.59	12.38^c^	23.61	34.75^b^
	Nar	34.69^c^	50.98	57.30	38.35^b^	52.28^c^	61.67^b^	15.88^c^	35.24	43.69	13.72^c^	27.04	35.25^b^
	Flavo	37.73^b^	48.50	61.64	40.69^a^	53.35^b^	62.79^a^ ^b^	20.83^b^	34.77	51.39	17.90^b^	27.64	44.03
**SEM^e^**	-	0.579	0.854	0.546	0.724	0.791	1.100	1.144	0.972	0.689	0.975	1.748	1.158
***Substrates***													
** LF**	-	27.93	41.99	53.65	32.10	45.00	55.46	16.47	33.30	47.18	12.40	25.57	39.49
** LF-S**		39.36	51.54	60.67	39.91	53.20	62.26	22.36	37.41	49.38	19.51	30.52	41.81
***Additives***													
** Con**	-	36.48	49.78^a^	59.01^a^	37.09	50.23	59.93	23.75	38.68^a^	50.74^a^	19.77	32.06^a^	43.30
** DFM1x**	-	36.56	49.53^a^	58.99^a^	37.57	50.75	60.48	23.29	38.89^a^	51.57^a^	20.41	32.10^a^	43.52
** DFM5x**	-	36.17	48.69^a^	58.29^a^	37.10	50.37	59.88	23.39	38.83^a^	50.76^a^	20.04	31.74^a^	43.19
** Mon**	-	30.59	43.06^b^	54.55^b^	33.43	46.49	56.32	14.24	29.86^c^	43.48^b^	10.19	21.63^d^	35.62
** Nar**	-	29.32	45.70^c^	53.69^b^	33.89	46.86	56.65	13.56	32.95^b^	42.81^b^	10.15	24.44^c^	34.93
** Flavo**	-	32.75	43.86^b^	58.42^a^	36.96	49.91	59.91	18.27	32.92^b^	50.31^a^	15.19	26.33^b^	43.34
***P-value***													
** Substrate**	-	<.0001	<.0001	<.0001	<.0001	<.0001	<.0001	<.0001	<.0001	<.0001	<.0001	<.0001	0.0002
** Additive**	-	<.0001	<.0001	<.0001	<.0001	<.0001	<.0001	<.0001	<.0001	<.0001	<.0001	<.0001	<.0001
** Subs × Add^e^**		0.0222	0.6804	0.8338	0.0472	0.0001	0.0004	0.0483	0.0676	0.1907	0.0432	0.3505	0.0060
***DFM orthogonal effect***													
** Linear**		0.5156	0.1910	0.1481	0.5515	0.9020	0.5768	0.7687	0.8848	0.6422	0.9332	0.6828	0.8280
** Quadratic**		0.7960	0.9674	0.8059	0.0654	0.1462	0.1948	0.5922	0.7693	0.1596	0.4420	0.9025	0.7900

a LF—low forage quality; LF-S—low forage quality plus protein-energetic supplement.

b Con: diet control without additive; Bovacillus 1x (DFM1x); Bovacillus 5x (DFM5x); Monensin (Mon); Narasina (Nar) and Flavomycin (Flavo).

c The *in vitro* organic matter was calculated according to Menke and Steingass (1988): IVOMD%= 16.49 + 0.9042GP + 0.0492CP + 0.0387TA.

d Least square means with different superscripts on the same column differ at *P *≤ 0.05.

e Substrate × additive interaction.

When the effect of feed additives was evaluated separately, IVDMD at 48 and 96 h was not altered (*P* > 0.05) by the inclusion of DFM (DFM *vs* Con); however, Mon and Nar reduced IVDMD compared with Con (*P* < 0.01). Flavo also reduced IVDMD at 48 h compared with Con, DFM1x and DFM5x (*P* < 0.01), but did not differ from Con, DFM1x, and DFM5x at 96 h (*P* > 0.05). Similar results were observed for IVNDFD (48 and 96 h) and IVADFD at 48 h.

#### NH3-N, pH and microbial biomass yield

There were significant interactions between substrates and feed additives (*P* < 0.05) for NH_3_-N at 24, 48, and 96 h of fermentation. No further interactions (*P* > 0.05) were reported for ruminal pH and microbial biomass yield (Table 8).

**Table 8.** *In Vitro* ruminal ammonia-N (NH_3_-N, mg/dL*)*, pH, and microbial biomass yield (MBY) of low-quality forage-based diets with different additives (exp. 2).

**Substrates** ^a^	Additives^b^	NH_3_-N, mg/dL			pH			MBY, mg/kg
		24 h	48 h	96 h	24 h	48 h	96 h	
**LF**	Con	0.55^b^	3.12^b^	5.58^a^ ^b^	6.88	6.86	6.80	374.05
	DFM1x	0.61^b^	3.48^a^ ^b^	5.09^a^ ^b^	6.87	6.86	6.81	372.49
	DFM5x	0.74^b^	3.29^a^ ^b^	4.68^b^	6.88	6.86	6.81	352.31
	Mon	1.56^a^	2.37^c^	2.69^c^	6.88	6.84	6.78	372.05
	Nar	1.33^a^	3.25^a^ ^b^	6.13^a^	6.87	6.85	6.77	367.80
	Flavo	0.75^b^	3.84^a^	4.68^b^	6.86	6.84	6.77	374.12
**LF-S**	Con	2.13^c^ ^d^	7.72^b^	8.15^b^	6.84	6.84	6.80	373.09
	DFM1x	2.15^c^ ^d^	6.62^c^	9.72^a^	6.84	6.82	6.80	367.56
	DFM5x	2.02^d^	9.53^a^	10.55^a^	6.85	6.83	6.80	368.68
	Mon	2.50^c^	8.17^b^	7.51^b^	6.85	6.82	6.76	378.89
	Nar	4.30^a^	9.70^a^	10.84^a^	6.86	6.83	6.78	353.48
	Flavo	3.27^b^	9.05^a^	10.24^a^	6.86	6.83	6.78	371.26
**SEM^d^**	-	0.146	0.245	0.459	0.013	0.012	0.018	8.578
***Substrates***								
** LF**	-	0.92	3.22	4.81	6.87	6.85	6.79	368.80
** LF-S**		2.73	8.46	9.50	6.85	6.83	6.79	368.83
***Additives***								
** Con**	-	1.34	5.42	6.86	6.86	6.85	6.80^a^	373.57
** DFM1x**	-	1.38	5.05	7.40	6.85	6.84	6.80^a^	370.02
** DFM5x**	-	1.38	6.41	7.61	6.86	6.85	6.80^a^	360.50
** Mon**	-	2.03	5.27	5.10	6.87	6.83	6.77^b^	375.47
** Nar**	-	2.82	6.47	8.48	6.87	6.84	6.77^b^	360.64
** Flavo**	-	2.01	6.44	7.46	6.86	6.83	6.77^b^	372.69
***P-value***								
** Substrate**	-	<.0001	<.0001	<.0001	0.0003	0.0003	0.7680	0.9958
** Additive**	-	<.0001	<.0001	<.0001	0.7132	0.3961	0.0021	0.2895
** Subs × Add^d^**		<.0001	<.0001	0.0067	0.5076	0.9575	0.7265	0.5513
***DFM orthogonal effect***								
** Linear**		0.8138	<.0001	0.1251	0.5400	0.8069	0.5630	0.1029
** Quadratic**		0.8176	0.0107	0.3066	0.3832	0.3768	0.6135	0.8999

a LF—low forage quality; LF-S—low forage quality plus protein-energetic supplement.

b Con: diet control without additive; Bovacillus 1x (DFM1x); Bovacillus 5x (DFM5x); Monensin (Mon); Narasina (Nar) and Flavomycin (Flavo).

c Least square means with different superscripts on the same column differ at *P *≤ 0.05.

d Substrate × additive interaction.

When the substrate was LF, supplementation with Mon and Nar increased NH_3_-N at 24 h compared with the other treatments (*P* < 0.01), but they did not differ (*P* > 0.05) from each other. However, when the substrate was LF-S, Nar and Flavo increased NH_3_-N compared with Con, DFM1x, DFM5x, and Mon (*P* > 0.0001), with Nar being also greater than Flavo, whereas Mon, DFM1x, and DFM5x did not differ from Con (*P* > 0.05; Table 8).

At 48 h, Flavo increased NH_3_-N compared with Con and Mon when LF was the substrate (*P* < 0.01), but did not differ from DFM1x, DFM5x, and Nar (*P* > 0.05). Mon decreased NH_3_-N compared with Con (*P* *<* 0.01; Table 8). However, when LF-S was used, Mon did not modify (*P* > 0.05) NH_3_-N compared with Con, whereas DFM1x decreased NH_3_-N compared with Con and the other treatments (*P* < 0.01). On the other hand, DFM5x, Nar, and Flavo had higher values compared with the other treatments (*P* < 0.01), but they did not differ from each other (*P* > 0.05).

At 96 h, when substrate was LF, NH_3_-N for DFM1x, DFM5x, Nar, and Flavo did not differ (*P* > 0.05) from Con, with Nar being higher (*P* < 0.01) than DFM5x and Flavo. The Mon treatment reduced NH_3_-N compared with all treatments (*P* < 0.01; Table 8). When the substrate was LF-S, DFM1x, DFM5x, Nar, and Flavo increased NH_3_-N compared with Con and Mon (*P* < 0.01), which did not differ from each other.

Ruminal pH at 24 and 48 h were ower for LF-S than LF (*P* < 0.01; Table 8). At 24 and 48 h, additives did not affect pH (*P* > 0.05; Table 8). However, at 96 h of incubation, DFM1x and DFM5x did not modify (*P* > 0.05) the pH compared with Con, but was greater (*P* < 0.01) when compared with Mon, Nar, and Flavo, that did not differ from each other (Table 8).

#### VFA

No substrate × feed additive interactions were observed for VFA profile (*P* > 0.05; Table 9). Overall, LF-S increased total VFA, propionate, and butyrate, and decreased isobutyrate, valerate, isovalerate, and acetate: propionate ratio compared with LF (*P* < 0.05).

**Table 9.** *In Vitro* total volatile fatty acids (VFA, mmol/L) and VFA profile (mmol/100 mmol) of low-quality forage-based diets with different additives (exp. 2).

**Item** ^a^	Total VFA	VFA profile, mmol/100 mmol^b^						
		Acet	Prop	But	Isobut	Val	Isoval	Acet: prop
***Substrates***								
** LF**	41.16	63.85	19.73	8.16	2.78	2.65	2.84	3.25
** LF-S**	45.53	63.03	20.96	8.50	2.52	2.43	2.56	3.04
**SEM**	2.149	0.340	0.358	0.123	0.094	0.097	0.079	0.069
***Additives***								
** Con**	44.56	64.32^a^	19.26^b^ ^c^	8.67^a^	2.60	2.49	2.66	3.37^a^
** DFM1x**	43.85	64.37^a^	19.13^c^	8.69^a^	2.62	2.52	2.68	3.38^a^
** DFM5x**	44.14	63.34^a^ ^b^	19.88^b^ ^c^	8.84^a^	2.67	2.55	2.72	3.19^a^
** Mon**	42.51	62.42^b^	21.94^a^	7.76^b^	2.64	2.54	2.70	2.86^b^
** Nar**	42.63	62.38^b^	21.83^a^	7.85^b^	2.66	2.58	2.69	2.87^b^
** Flavo**	42.37	63.81^a^ ^b^	20.04^b^	8.15^b^	2.69	2.58	2.74	3.19^a^
**SEM**	2.299	0.539	0.434	0.175	0.106	0.109	0.094	0.092
***P-value***								
** Substrate**	<.0001	0.057	<.0001	0.0100	<.0001	<.0001	<.0001	0.0013
** Additive**	0.4886	0.0234	<.0001	<.0001	0.8933	0.8743	0.9635	<.0001
** Subs × Add^d^**	0.1121	0.7019	0.0672	0.0673	0.1626	0.2596	0.271	0.439
***DFM orthogonal effect***								
** Linear**	0.8821	0.127	0.0852	0.3906	0.3945	0.4807	0.5615	0.0597
** Quadratic**	0.6292	0.7177	0.5135	0.9234	0.8793	0.8481	0.9204	0.6064

a LF—low forage quality; LF-S—low forage quality plus protein-energetic supplement; (Con): diet control without additive; Bovacillus 1x (DFM1x); Bovacillus 5x (DFM5x); Monensin (Mon); Narasina (Nar) and Flavomycin (Flavo); SEM—Standard error of mean; Subst x add: effects ot the substrate and additives.

b Acet: acetate, Prop: Propionate, But: Butyrate, Isobut: Iso-butyrate, Val: Valerate, Isoval: Iso-valerate, Acet: Prop: Acetate: Propionate.

c Least square means with different superscripts on the same column differ at *P *≤ 0.05.

d Substrate × additive interaction.

There was no significant difference (*P* > 0.05) for total VFA, isobutyrate, valerate, and isovalerate following additive inclusion (Table 9). Moreover, Mon and Nar decreased (*P* *=* 0.02) acetate compared with Con and DFM1x, without further differences from other treatments (*P* > 0.05). Inversely, Mon and Nar increased (*P* *<* 0.01) propionate compared with Con and other treatments. Inversely, DFM1x, DFM5x, and Flavo did not differ from Con (*P* > 0.05). Additionally, Mon and Nar decreased (*P* < 0.01) acetate: propionate ratio compared with Con and other treatments, which did not differ from each other (*P* = 0.05; Table 9).

## Discussion

The objective of these studies was to evaluate the effects of a *Bacillus*-based DFM on *in vitro* rumen fermentation parameters, gas production kinetics, and fiber digestibility when compared with other feed additives under two forage-based dietary scenarios. It was hypothesized that the DFM, due to its enzymatic activity (Rhayat et al. 2019; Schallmey et al. 2004), would enhance rumen fermentation and increase fiber digestibility in tropical forage-based diets, whereas the other additives (ionophores and non-ionophore) were expected to either maintain or reduce fiber digestibility possibly due to reduced fibrolytic activity by rumen microbes (Brutti et al. 2019; Ormond et al. 2025).

Forage plays a central role in the nutrition of ruminants, especially those raised in the tropics (Beauchemin et al. 2003; Cooke et al. 2020), as well as for cow-calf and stocker operations. The challenges associated with grazing cattle production are related to the variability of forage availability and nutritional profiles throughout the year, which directly impacts the efficiency and sustainability of the production system. Regardless of the season, forages often do not provide the required amount of dietary nutrients to meet the nutritional needs of grazing animals (Almeida et al. 2022; Detmann et al. 2014), indicating the need for a supplementation strategy (McDowell and Arthington 2005). Indeed, insufficient nutrients, especially the low concentrations and availability of CP and digestible energy, are thought to be responsible for limiting microbial growth in the rumen, with a negative impact on fiber digestion (Detmann et al. 2014; Leng 1990). This scenario becomes even more critical when medium- to low-quality warm-season forages are considered (Bohnert et al. 2011). Altogether, these factors have a direct influence on the performance of the grazing beef cattle herd by compromising both voluntary feed intake and nutrient digestibility (Dhakal et al. 2023). Therefore, a supplementation program would be the most advantageous and recommended strategy to compensate for the nutritional limitations of animals reared in grazing tropical pastures (Paulino et al. 2006).

The results of our *in vitro* study indicate that the inclusion of energy-protein supplements in tropical forage-based diets, whether medium- or low-quality, enhances nutrient utilization. This was evidenced by the increased gas production and fiber digestibility. However, the magnitude of the response to the energy-protein supplement was more pronounced in the low-quality forage (Almeida et al. 2022; Detmann et al. 2014).

The forage used in Exp. 1 (medium-quality) contained 90.64 g CP/kg DM (Table 1), a higher content than that recommended by Van Soest (1994) and Lazzarini et al., (2009), who suggested a concentration of 70-80 g CP/kg DM to avoid limitations in microbial growth and efficient digestion of the fibrous carbohydrates of the forages. However, there is a characteristic nutritional imbalance in medium-quality tropical pastures, which is characterized by a relative excess of energy in relation to available CP (Detmann et al. 2014). These latter authors suggested that the rumen N balance becomes positive when the NH_3_-N concentrations exceed 9.7 mg/dL and is maximized when exceeding 15.9 mg/dL. In the current study, the NH_3_-N only exceeded 10 mg/dL when the energy-protein supplement was used at 48 and 96 h of incubation, suggesting that we achieved the minimum threshold proposed by Detmann et al. (2014), but did not optimize rumen nutrient synchrony.

In Exp. 2, which evaluated a more challenging scenario of the year (dry season), the substrate contained only 30 g CP/kg DM, a concentration well below the recommended values ­(Detmann et al. 2014). Nitrogen deficiency in the rumen is the main factor limiting digestibility of low CP forage, being the most important nutrient to optimize fermentative digestion of forage (Almeida et al. 2022; Detmann et al.2014; Leng 1990; Satter and Slyter 1974; Van Soest 1994). A positive effect of N supplementation on fiber degradation is only supposed to be observed with NH_3_-N concentrations reach up to 8 mg/dL (­Detmann et al. 2014). Supplementation with EPS increased the CP content of diet to 75.5 g/kg DM and NH_3_-N concentrations to 8.5 and 9.5 at 48 and 96 h, respectively. However, when the forage was not supplemented, NH_3_-N dropped to levels below 5 mg/dL. In this study, the inclusion of EPS in the low-quality forage increased NDF digestibility by 35.7 and 12.3% at 24 and 48 h, respectively.

On the other hand, feed additives have been studied due to their potential in altering rumen fermentation and nutrient digestibility, thereby improving the energy efficiency of the beef cattle herd (Russell and Strobel 1989; Tedeschi et al. 2003; Limede et al. 2021; Terry and Beauchemin 2025). However, most of the research with feed additives, more specifically ionophore and non-ionophore compounds, available in the literature has been conducted in animals fed high-concentrate diets (Tedeschi et al. 2003; Duffield et al. 2008; Limede et al. 2021).

With increasing concern over the use of antimicrobials in livestock production, some classes of feed additives have been viewed as a “natural” alternative to traditional antibiotics. In this sense, the use of direct-feed microbials (DFM), also known as probiotics—live microorganisms that confer health benefits—has gained attention due to their positive effects on the health and performance of dairy and beef cattle (Magalhães et al. 2024; McAllister et al. 2011; Cappellozza et al. 2023). *Bacillus spp*. is one of the DFM options available for ruminants, being classified as gram-positive, catalase-positive, spore-forming, aerobic, and facultative anaerobic bacteria (Luise et al. 2022).

The results of our *in vitro* study indicate that DFM, monensin, narasin, and flavomycin modulate ruminal fermentation in distinct, diet-dependent ways. The inclusion of DFM5x increased total gas production in the initial timepoints of fermentation (12 and 24 h), particularly when the forage was of medium quality, but no effects were observed on fiber digestibility. The absence of differences in dry matter and fiber digestibility with DFM inclusion was not expected, as *Bacillus* species are known to secrete a wide and diverse array of enzymes—including fibrolytic, amylolytic, proteolytic, and lipolytic enzymes—that could enhance nutrient digestion (Schallmey et al. 2004; Luise et al. 2022; Cappellozza et al. 2023; Lopez et al. 2024). Additionally, *Bacillus* cultures can supply metabolites such as vitamins, glucose, lactate, malate, formate, succinate, and aspartate once lysis occurs (Qiao et al. 2010). Recent studies have evaluated the inclusion of single or combined strains of DFM. Pan et al., (2022) evaluated the effects of the same *B. licheniformis* 809 and *B. subtilis* 810 on *in vitro* DM and NDF digestibility of Australian forages and observed improvements of DM and NDF digestibility in most of the forages evaluated. Cappellozza et al., (2023) evaluated six single fiber-based feedstuffs and nine commercial dairy TMR and found that the inoculation with the same *Bacillus* spp. used herein improved mean *in vitro* gas production, dry matter, and neutral detergent fiber digestibility, but no differences were observed *in vitro* starch digestibility. Similar findings were reported by Silva et al., (2024) using low-quality tropical grass as a substrate, however the proportional improvement on IVOMD was smaller than in prior studies (Pan et al. 2022; Cappellozza et al. 2023).

Previous *in vitro* studies using *B. licheniformis* 809 and *B. subtilis* 810 also reported improved gas production at 24 and 48 h for forage substrates or at 48 h when commercial dairy TMRs were evaluated (Cappellozza et al. 2023). Moreover, as *Bacillus* spp. secrete enzymes directly into the feedstuff, bacterial attachment to the substrate must occur in order to start nutrient digestion. Recently, Limede et al. (2025) reported improvements on *in situ* NDF and ADF degradability when feedlot-based TMRs were incubated in rumen-fistulated beef cows fed *B. licheniformis* 809 and *B. subtilis* 810, suggesting a beneficial effect of *Bacillus* spp. on initial adherence to feed particles and consequent reduction in lag time. However, a similar response was not observed in the current study, either in low- or mid-quality forages. Additional studies are warranted to understand the potential synergy between *Bacillus* spp., substrate adherence, and enzymatic secretion, which may contribute to a greater nutrient utilization in beef and dairy cattle.

On the other hand, the ionophores (monensin and narasin) significantly decreased fiber digestibility, regardless of the forage quality and/or energy-protein supplementation. Among ionophores, monensin is the most extensively researched and widely used in beef cattle diets to enhance the energy efficiency of cattle (Duffield et al. 2012; Terry and Beauchemin 2025). Ionophores are a class of compounds with diverse chemical structures with selective inhibition of gram-positive over gram-negative bacteria, thereby increasing ruminal propionate production and reducing acetate proportions, with a consequent reduction in feed intake and improvement in feed conversion, as well as suppression of ruminal protein degradation (Russell and Strobel 1989; Tedeschi et al. 2003; Appuhamy et al. 2013). However, many of these inferences were obtained from high-grain diets. The influence of forage quality on the productive response to feed additives in beef cattle has not been extensively studied (Bretschneider et al. 2008). Given its mode of action, the use of ionophores in high-forage diets such as those based on tropical forages where the primary goal is to enhance fiber digestion should be approached with caution. The reduction in DM and NDF digestibility observed with medium and low-quality forage following monensin inclusion may be attributed to its inhibitory effect on gram-positive, fibrolytic bacteria, such as *Ruminococcus albus, Ruminococcus flavefaciens, and Butyrivibrio fibrisolvens* (Anassori et al. 2012). Supporting our findings, Brutti et al., (2019) observed that *in vitro* DM and NDF digestibility decreased with monensin supplementation in both N-fertilized and non-fertilized *Urochloa brizantha* cv. Marandu.

Likewise, narasin inclusion also decreased fiber digestibility in both forage-based experiments. The mode of action of the narasin in the rumen is similar to that of monensin (Marques and Cooke 2021), and in recent years, narasin has been increasingly studied in high-forage diets (Miszura et al. 2018; Polizel et al. 2020; Limede et al. 2021). Limede et al., (2021) investigated the inclusion of narasin, salinomycin, and flavomycin in forage-based diets and reported no significant effects on apparent digestibility of nutrients. Similarly, Polizel et al., (2020) found no effect of narasin supplementation on total DMI or nutrient digestibility to beef steers receiving a high-forage diet. The effects of ionophores (monensin and narasin) on nutrient digestibility appear to be variable (Spears 1996; Polizel et al. 2020). The efficacy of ionophores may be influenced by doet composition, which can affect the concentration and balance of cations (e.g., Na^+^ to K^+^) in ruminal fluid (Russell and Strobel 1989; Wilman et al. 1994; Bretschneider et al. 2008).

The non-ionophore evaluated in this study was flavomycin, a phosphoglycolipid antibiotic produced by *Streptomyces bambergiensis, S. geysirensis*, and *S. ederensis*, which may modify rumen fermentation by inhibiting peptidoglycan synthesis in the bacterial cell wall (Volke et al. 1997) and suppressing gram-positive bacteria, leading to a greater proportion of propionate (Edrington et al. 2003). Additionally, flavomycin may alter the ruminal protozoa population, which in turn might improve fiber digestion (Perry 2002). In the current study, as observed with DFM, flavomycin was effective in increasing gas production during the initial phase of incubation when the forage was of medium quality, but did not elicit a effect on fiber digestibility. However, when forage was of low-quality, flavomycin reduced *in vitro* fiber digestibility.

An important point is that most of these studies evaluating traditional feed additives supplementation used high-quality forage (frequently C3), with CP content above 7.0%. In these scenarios, CP does not appear to limit microbial growth and rumen function (Detmann et al. 2014). The literature is scarce concerning the effects of ionophores for cattle consuming high-forage diets of varying botanical composition and nutritive values (Bretschneider et al. 2008; Polizel et al. 2020). Ionophores might reduce ruminal proteolysis and consequently lower NH_3_-N (Rogers et al. 1997; Tedeschi et al. 2003). However, NH_3_-N concentrations below 8 mg/dL might limit microbial growth and ruminal fermentation (Detmann et al. 2014). In our study, monensin and narasin reduced NH_3_-N when forage was of medium-quality; however, narasin increased NH_3_-N compared to control when forage was low-quality. Polizel et al. (2020) showed that narasin reduced NH_3_-N with Tifton-85 haylage containing CP ranging from 6.7 to 12.4%. Conversely, Limede et al., (2021) did not find differences in NH_3_-N (forage CP = 16.0–18.9%) with narasin supplementation. Bell et al. (2017) also did not observe impact of monensin on NH_3_-N with forage with 13.1% CP and DDGS supplementation. On the other hand, the addition of *Bacillus* spp. increased rumen NH_3_-N in low-quality forage with supplementation (48 and 96 h), and in medium-quality with and without supplementation at 96 h. *Bacillus* spp. also secrete proteases (Luise et al. 2022) that can lead to a greater *in situ* CP degradation in the rumen (Limede et al. 2024). However, as previously mentioned, the scenario for both dietary situations were challenging in terms of N supply needed to maintain adequate nutrient synchrony (Hall and Huntington 2008) and, in this case, an increase in NH_3_-N with *Bacillus* spp. could be associated with enhanced N recycling and therefore, a greater supply of N to keep an adequate rumen function. However, this hypothesis would need to be further validated *in vivo*, particularly under conditions where N supply in the diet is limiting and/or offered infrequently.

Inclusion of ionophores (ie monensin) in ruminants’ diets frequently modifies rumen fermentation parameters with greater molar proportion of propionate, as well as reduced acetate and acetate: propionate ratio, thereby increasing the efficiency of energy metabolism (Tedeschi et al. 2003). The results of the current study support this statement with monensin and narasin regardless of the forage quality. Supporting our results, *in vivo* studies also reported an increased concentration of rumen propionate and reduced concentration of acetate when narasin was supplemented in forage-based diets (Miszura et al. 2018; Polizel et al. 2020; Limede et al. 2021). Conversely, the inclusion of the non-ionophore flavomycin did not change individual ruminal VFA production and proportion. Accordingly, *in vivo* studies did not observe changes VFA (Crossland at al. 2017; Limede et al. 2021). Despite the changes in VFA proportions, feed additives used in this study did not modify total VFA production. Therefore, the positive *in vitro* responses of the additives on VFA in forage diets is related to a shift in acetate: propionate ratio toward propionate, but not due to an increase in total VFA production. On the other hand, *Bacillus* spp. supplementation had limited effects on rumen VFA profile when a low-quality tropical forage was used as an *in vitro* substrate (Silva et al. 2024).

## Conclusion

Regardless of the forage quality, energy-protein supplementation showed a positive response in rumen fermentation and fiber digestibility of forage-based diets, however, the magnitude of this response was more pronounced low-quality forage. The inclusion *Bacillus*-based direct-fed microbial containing *Bacillus licheniformis* 809 and *Bacillus subtilis* 810 and flavomycin increased gas production in the initial hours of incubation, especially with medium quality forage, but did not improve *in vitro* dry matter or fiber digestibility, whereas ionophores had deleterious effects on the *in vitro* digestibility. In contrast, ionophores negatively affected *in vitro* digestibility, despite reducing acetate: propionate ratio. Given the increasing scrutiny over the use of antibiotics and potential antimicrobial resistance uprise in animal diets, our findings suggest that direct-fed microbial may be a viable alternative supplement to ionophores for forage-fed beef steers grazing *Urochloa brizantha* cv. Marandu without impairing ruminal fermentation. In our study, the direct-fed microbial supplementation provided similar results over the use of no additive, however further *in vivo* studies are warranted to evaluate the effects of feed additive inclusion in forage-based diets of varying quality.
